# P2Y_12_ Receptor Antagonist Clopidogrel Attenuates Lung Inflammation Triggered by Silica Particles

**DOI:** 10.3389/fphar.2020.00301

**Published:** 2020-03-18

**Authors:** Patricia Teixeira Santana, Tatiana Luna-Gomes, Marcos Vinicius Rangel-Ferreira, Augusto Shuiti Tamura, Carolyne Lalucha Alves Lima Da Graça, Mariana Nascimento Machado, Walter Araujo Zin, Christina Maeda Takiya, Debora Souza Faffe, Robson Coutinho-Silva

**Affiliations:** Institute of Biophysics Carlos Chagas Filho, Federal University of Rio de Janeiro, Rio de Janeiro, Brazil

**Keywords:** silica particles, silicosis, purinergic receptors, P2Y_12_ receptor, ADP, clopidogrel

## Abstract

Silicosis is an occupational lung disease caused by inhalation of silica particles. It is characterized by intense lung inflammation, with progressive and irreversible fibrosis, leading to impaired lung function. Purinergic signaling modulates silica-induced lung inflammation and fibrosis through P2X7 receptor. In the present study, we investigate the role of P2Y_12_, the G-protein-coupled subfamily prototype of P2 receptor class in silicosis. To that end, BALB/c mice received an intratracheal injection of PBS or silica particles (20 mg), without or with P2Y_12_ receptor blockade by clopidogrel (20 mg/kg body weight by gavage every 48 h) – groups CTRL, SIL, and SIL + Clopi, respectively. After 14 days, lung mechanics were determined by the end-inflation occlusion method. Lung histology was analyzed, and lung parenchyma production of nitric oxide and cytokines (IL-1β, IL-6, TNF-α, and TGF-β) were determined. Silica injection reduced animal survival and increased all lung mechanical parameters in relation to CTRL, followed by diffuse lung parenchyma inflammation, increased neutrophil infiltration, collagen deposition and increased pro-inflammatory and pro-fibrogenic cytokine secretion, as well as increased nitrite production. Clopidogrel treatment prevented silica-induced changes in lung function, and significantly reduced lung inflammation, fibrosis, as well as cytokine and nitrite production. These data suggest that inhibition of P2Y_12_ signaling improves silica-induced lung inflammation, preventing lung functional changes and mortality. Our results corroborate previous observations of silica-induced lung changes and expand the understanding of purinergic signaling in this process.

## Introduction

Silicosis is an occupational pneumoconiosis caused by inhalation of silica particles (free crystalline silicon dioxide), which remains a health problem for workers in many industries, including mining and civil construction (Kauppinen et al., 2000; Bhagia, 2012). New forms of exposure to silica particles are added to those classically implied as silica sources, such as dental supply factories using quartz fillers (de la Hoz et al., 2004), dental technicians exposed to airborne residuals of silica (Ergün et al., 2014), jewelry workers exposed to silica-containing chalk molds used in casting (Murgia et al., 2007), denim sandblasters (Bakan et al., 2011; Akgun, 2016), and fabricators of artificial-stone worktops (Hoy et al., 2018). Silica particle deposition in lung parenchyma leads to intense inflammatory response, followed by progressive and irreversible lung fibrosis. Depending on the dose, silica may produce acute (accelerated silicosis) or various forms of chronic silicosis (Borges et al., 2002; Castranova et al., 2002; Hnizdo and Vallyathan, 2003; Langley et al., 2004; Rimal et al., 2005). Both high-dose acute and low-dose chronic silica exposures induce granulomatous changes in the lungs. The risk of disease is related to lifetime cumulative exposure and to the amount of inhaled crystalline silica, which, in turn, depends on the concentration and the size of respirable particles, as well as on individual susceptibility (Leung et al., 2012).

Purinergic signaling has been studied during silicosis and various inflammatory contexts, where it contributes to inflammatory exacerbation (Burnstock and Kennedy, 2011; Idzko et al., 2014; Savio et al., 2018). P2 class receptors are activated by extracellular nucleotides, such as ATP and ADP, and are subdivided into two subfamilies: P2X ligand-gated ion channels, and P2Y G-protein-coupled receptors (Burnstock and Kennedy, 2011). We previously demonstrated a significant role for P2X7 receptor as a regulator of silica-induced lung changes. Silica-induced ATP release activates P2X7 receptor, leading to the production of reactive oxygen species (ROS), inflammasome activation, and IL-1β release (Moncao-Ribeiro et al., 2014). By contrast, the role of P2Y receptors in silica inflammation is less well understood.

P2Y_12_ receptor is mainly, but not exclusively, expressed on platelets. It mediates ADP-induced platelet aggregation, playing a central role in platelet biology (Kim and Kunapuli, 2011). More recently, P2Y_12_ expression has been described also in immune cells, such as monocytes (Micklewright et al., 2018), dendritic cells (Ben Addi et al., 2010), and T lymphocytes (Wang et al., 2004). Furthermore, blocking P2Y_12_ pathways alters T cell activation and changes the cell population (Vemulapalli et al., 2019). In the respiratory system, P2Y_12_ receptor appears to contribute to inflammatory response, participating in allergic and non-allergic processes (Paruchuri et al., 2009; Shirasaki et al., 2013; Suh et al., 2016), as well as autoimmune disease processes (Domercq et al., 2018). Therefore, to better understand the role of purinergic signaling in silica-induced lung inflammation, we investigated the participation of P2Y_12_ receptor in the onset of silicosis.

## Materials and Methods

### Experimental Group

This study was approved by the Ethics Committee of the Health Sciences Center, Federal University of Rio de Janeiro (IBCCF164). All animals received humane care according to the Guiding Principles in the Care and Use of Laboratory Animals approved by the Council of the American Physiological Society. Male Balb/c mice (20–30 g, *n* = 36) were anesthetized with isoflurane (Isoforine^®^, Cristália, São Paulo, Brazil; 99% purity) and randomly divided into three groups, intratracheally injected with: phosphate-buffered saline (PBS, 100 μL) (CTRL group) or 20 mg of silica particles (approximately 80% 1–5 μm, Sigma, Chemical Co., St. Louis, MO, United States) without (SIL) or with (SIL + Clopi) clopidogrel (Plavix^®^, Sanofi-Aventis, Paris, France; 99% purity) treatment (20 mg/kg body weight by gavage each 48 h for 14 days). Animals were analyzed 14 days after PBS or silica administration.

### Pulmonary Mechanics

Pulmonary mechanics were determined as previously described (Moncao-Ribeiro et al., 2014). Briefly, animals were sedated (diazepam 1 mg *i.p.* Valium^®^, Roche, Basel, Switzerland; 99% purity), anesthetized (pentobarbital sodium 20 mg/kg body weight *i.p.*, Nembutal^®^, Merck, Beijing, China; 99% purity), paralyzed (pancuronium bromide 0.1 mg/kg body weight *i.v.* Pancuron^®^, Cristália, São Paulo, Brazil; 99% purity), and mechanically ventilated (Samay VR15, Universidad de la República, Montevideo, Uruguay) with 100 breaths/min, tidal volume of 0.2 mL, flow of 1 mL/s, and positive end-expiratory pressure of 2.0 cmH_2_O. The anterior chest wall was surgically removed, airflow (V′) was measured using a pneumotachograph (1.5-mm ID; length = 4.2 cm, distance between side ports = 2.1 cm) connected to the tracheal cannula, lung volume was obtained digital integration of the flow signal. The pressure gradient across the pneumotachograph and transpulmonary pressure were determined using Validyne MP-45-2 differential pressure transducers (Engineering Corp., Northridge, CA, United States). Lung airway resistance and stress relaxation/viscoelastic properties [resistive (ΔP1), viscoelastic/inhomogeneous (ΔP2), and total (ΔPtot) pressures, respectively], as well as lung elastance and ΔE, were determined by the end-inflation occlusion method, as previously described (Bates et al., 1985).

### Pulmonary Histology and Histomorphometry

To verify silica-induced pulmonary lesions an additional group of mice subjected to the same experimental protocol described above was used. Fourteen days after PBS or silica injection, the left lungs were collected, fixed with 4% buffered formaldehyde solution, dehydrated, and embedded in paraffin. Sections (4-μm – thick) were cut and stained with hematoxylin-eosin for the description of qualitative alterations in the lung structure. Picrosirius red staining was performed to analyze collagen deposition. For histomorphometry quantifications, a computer-assisted image analysis system comprising a Nikon Eclipse E-800 microscope connected to a computer with a digital camera (Evolution, Media Cybernetics, Bethesda, MD, United States) coupled to Q-Capture 2.95.0 software (Silicon Graphic Inc., Milpitas, CA, United States) was used. High-quality photomicrographs (2048 × 1536 pixel buffer) were captured from non-overlapping lung areas. Data acquisition and analysis were done without knowledge of the animal groups in all cases, by the same observer.

Twenty high-quality images of silicotic nodules at ×10 objective lens were analyzed per animal. The surface density of silicotic nodules was calculated as follows: (total nodular area × 100)/total image area. Neutrophil quantification in lung parenchyma was determined in 16 images/animal at ×40 objective lens (3–5 animals/group). Results were expressed as the total number of neutrophils/histological field. Collagen fiber deposition in lung parenchyma was quantified across 20 random non-coincident fields (×10 objective lens). Results were expressed as the percentage of surface density/total image area.

### Nitric Oxide and Cytokine Measurements

The right lungs (from the same animals used for histological study) were macerated for nitric oxide (NO) and cytokine measurements in lung tissue homogenates. NO production was evaluated according to Griess method (Green et al., 1982), and fluorescence was measured at 570 nm wavelength (SpectraMax M, Molecular Devices, San Jose, CA, United States).

Cytokine concentrations (IL-1β, IL-6, TNF-α, and TGF-β) were determined by ELISA, with a detection limit of 50 pg/mL (R&D Systems, Minneapolis, MN, United States).

### Statistical Analysis

One-way ANOVA, followed by Bonferroni post-test, was used to assess differences among groups. Student’s *t*-test for independent samples, Chi-square test or Wilcoxon–Mann–Whitney test were applied whenever applicable. The level of significance was set at 5%.

## Results

### Treatment With P2Y_12_ Receptor Antagonist Reduced Weight Loss and Protected Animals From Death

**FIGURE 1:**
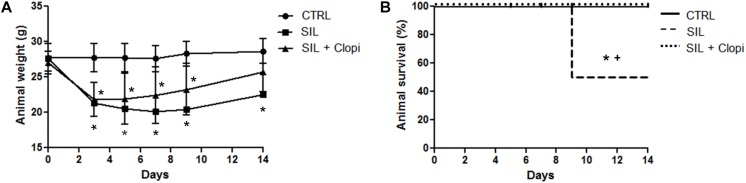
P2Y_12_ inhibition reduces silica-induced weight loss and prevents animal death. Body weight **(A)** and survival **(B)** were monitored along 14 days after PBS or silica (20 mg) injection without or with clopidogrel treatment (20 mg/kg) (CTRL, SIL, and SIL + Clopi groups, respectively). Values are mean ± SD of *n* = 5–6 animals/group. *p < 0.05 compared with CTRL; ^+^*p* < 0.05 in relation to SIL + Clopi.

Silica exposure induced weight loss along the 14 days after instillation, followed by a 50% reduction in survival rate. Clopidogrel treatment reduced weight loss (Figure 1A) and significantly improved animal survival (Figure 1B).

### P2Y_12_ Receptor Inhibition Led to Minor Changes in Lung Mechanics During Silicosis

**FIGURE 2:**
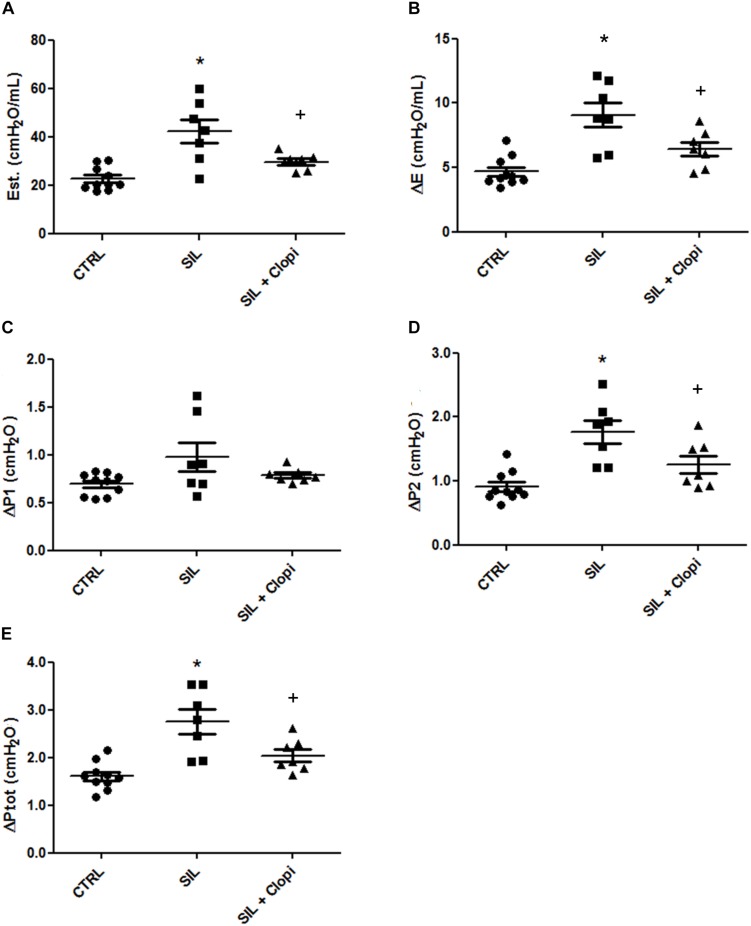
P2Y_12_ inhibition reduces silica-induced changes in lung mechanics. Lung static elastance (Est, **A**); viscoelastic component of elastance (ΔE, **B**); resistive (ΔP1, **C**), viscoelastic/inhomogeneous (ΔP2, **D**), and total (ΔPtot, **E**) pressures determined 14 days after PBS or silica (20 mg) injection without or with clopidogrel treatment (20 mg/kg) (CTRL, SIL, and SIL + Clopi groups, respectively). Values represent mean + SEM of 5–6 animals/group (10 determinations per animal). *p < 0.05 in relation to control (CTRL); ^+^*p* < 0.05 in relation to silica (SIL).

As previously reported (Faffe et al., 2001; Moncao-Ribeiro et al., 2014), silica caused lung functional changes, increasing all lung mechanical parameters in relation to control. Inhibition of the P2Y_12_ receptor by clopidogrel broadly improved lung function, including its resistive, elastic, and viscoelastic components (Figure 2).

### P2Y_12_ Receptor Inhibition Reduced Cellular Infiltration in Lung Parenchyma

**FIGURE 3:**
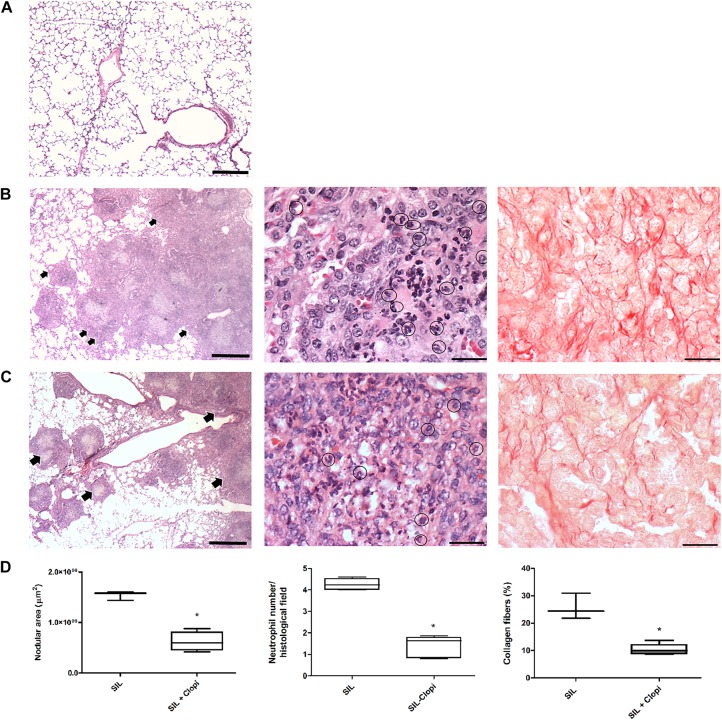
P2Y_12_ inhibition reduces silicotic lung inflammation and fibrosis. Representative lung parenchyma photomicrographs 14 days after PBS **(A)** or silica (20 mg) injection without **(B)** or with **(C)** clopidogrel treatment (20 mg/kg) (CTRL, SIL, and SIL + Clopi groups, respectively). Hematoxylin-eosin staining (left and central columns) and picrosirius staining (right column). **(D)** Quantification of nodular area, neutrophil infiltration, and collagen fibers (left, central, and right columns, respectively). Box plots show median values of 3–5 animals/group (16–20 images/animal) with respective minimum to maximum values. *p < 0.05 in relation to silica (SIL). Arrows show inflammatory infiltrates. Bars: 1000 μm (left column), 50 μm (central and right columns).

Silica administration led to intense and diffuse lung parenchyma infiltration of inflammatory cells, such as neutrophils, granulomatous nodular formation, and collagen fibers deposition (Figures 3A,B), as previously described (Faffe et al., 2001; Moncao-Ribeiro et al., 2014). In contrast, clopidogrel-treated mice showed preserved areas of lung parenchyma with morphological delimitation of the alveolar septa, significantly fewer neutrophil infiltration, as well as collagen fibers deposition compared with the SIL group (Figures 3C,D).

### P2Y12 Receptor Inhibition Reduced Silica-Induced Pro-Inflammatory and Pro-Fibrogenic Cytokine Secretion, as Well as Nitric Oxide Production

**FIGURE 4:**
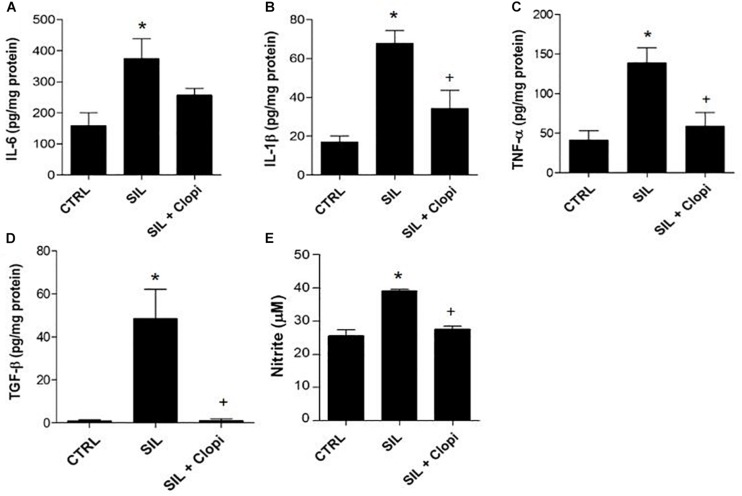
P2Y_12_ inhibition reduces silica-induced pro-inflammatory and pro-fibrotic cytokines, as well as nitrite production in lung parenchyma macerates. **(A)** IL-6, **(B)** IL-1β, and **(C)** TNF-α pro-inflammatory, and **(D)** TGF-β pro-fibrotic cytokines by ELISA. **(E)** nitrite production. Values are mean + SEM of 5–6 animals/group determined 14 days after PBS or silica (20 mg) injection without or with clopidogrel treatment (20 mg/kg) (CTRL, SIL and SIL + Clopi groups, respectively). *p < 0.05 in relation to control (CTRL); ^+^*p* < 0.05 in relation to silica (SIL).

Silica instillation induced pro-inflammatory and pro-fibrogenic cytokine production in lung parenchyma – such as IL-6, IL-1β, TNF-α, and TGF-β – as well as increased nitrite production (Figure 4). Conversely, clopidogrel treatment significantly reduced silica-induced cytokine and nitrite secretion (Figure 4).

## Discussion

Purinergic signaling has been implicated in the development of several inflammatory diseases. We have previously demonstrated the role of P2X7 in silicosis (Moncao-Ribeiro et al., 2014; Luna-Gomes et al., 2015), an irreversible and progressive lung fibrotic disease characterized by long-lasting inflammation. The present study expands the understanding of purinergic signaling in silica-induced lung inflammation, evaluating the role of P2Y_12_ receptor in a well-established murine model of silicosis. P2Y_12_ receptor blockage prevented silica-induced lung inflammatory changes, improving lung function and animal survival. These results demonstrate that P2Y_12_ signaling also participates in silicosis onset.

Nucleotides, such as ATP and ADP, are secreted during inflammation and bind to purinergic receptors, stimulating immune system cells in a paracrine and autocrine way (Idzko et al., 2014). Purinergic signaling is mediated by P2X (ATP-gated cation channels) and P2Y (G-protein-coupled) receptors (Burnstock, 2007; Shirasaki et al., 2013). P2X7 receptors have been shown important for leukocyte biology (Gu et al., 2000), as well as for silica-induced inflammation through NLRP3 inflammasome activation and IL-1β production (Moncao-Ribeiro et al., 2014; Luna-Gomes et al., 2015). On the other hand, P2Y_12_ receptors, the prototype of P2Y subfamily, are important for platelet biology (Buvinic et al., 2002; Guns et al., 2005). Although mainly expressed on platelets, P2Y_12_ expression was recently described in other cells of the immune system as well (Wang et al., 2004; Ben Addi et al., 2010; Junger, 2011; Micklewright et al., 2018). Indeed, P2Y_12_ receptor blockage influences T cell activation and cell proliferation. The effect of ADP is specific for CD4 and CD8 T cells, while P2Y_12_ antagonism alters these effects, suggesting functional expression of P2Y_12_ on T cells (Vemulapalli et al., 2019). We showed previously that silica-induced inflammation increased macrophage, neutrophil, dendritic cell, as well as lymphocyte infiltration (CD4^+^ and CD8^+^) in lung parenchyma (Moncao-Ribeiro et al., 2014). The present results now underline important participation of P2Y_12_ signaling in inflammatory cell recruitment on the onset of the silicotic process. Our data support also recent evidence of the importance of P2Y_12_ in biological functions of other immune cells rather than platelets. Recent data demonstrated a regulatory role for P2Y_12_ receptor in regulating neutrophil influx into the lung during sepsis (Liverani et al., 2016). P2Y12 receptor antagonism also reduced inflammation in other inflammation models, including pancreatitis, ischemia-reperfusion, and LPS-induced lung injury (Hackert et al., 2009; Harada et al., 2011; Liu et al., 2011). It is worth note, however, that the P2Y_12_ inhibitor clopidogrel – successfully used as antiplatelet medication to prevent thrombus formation in those at high risk – may also have P2Y_12_ independent effects during inflammation, and neutrophils are the most likely target (Liverani et al., 2016). Therefore, we cannot exclude an additional direct effect of clopidogrel on reduced inflammation observed after P2Y_12_ inhibition.

The immune physiopathology of silicosis involves the activation of inflammatory cells, especially alveolar macrophages. It has been shown that these cells contribute to increased lung oxidant secretion, as well as other inflammatory mediators, including interleukin 1β and tumor necrosis factor-alpha (TNF-α) (Jagirdar et al., 1996). NO is associated with inflammation and damage in asthma and LPS-induced inflammation (Belvisi et al., 1995; Moncao-Ribeiro et al., 2011; Liverani et al., 2014). NO also plays a crucial role in murine silicosis. Silica particle exposure activates macrophages to release NO (Moncao-Ribeiro et al., 2011). *In vivo* studies showed that mice exposed to silica develop exacerbated lung inflammation, while iNOS-deficient mice are more resistant to silica-induced inflammation (Srivastava et al., 2002). Previous studies showed that silica particle exposure induces TNF-α, IL-1β, and IL-6 secretion in lung parenchyma in a time-dependent manner (Vanhee et al., 1995; Moncao-Ribeiro et al., 2014). IL-1β is associated with cell recruitment, leading to neutrophil and eosinophil infiltration into lung tissue (Sims and Smith, 2010). In the context of silicosis, the lysis of alveolar macrophage releases cellular components into the extracellular environment, including IL-1β, promoting the recruitment of inflammatory cells into alveoli and endothelial walls (Moncao-Ribeiro et al., 2014). Purinergic signaling participates in IL-1β secretion by macrophages, as well as in NO production through P2X7 activation, as previously demonstrated by our group. On the other hand, P2Y_12_ receptor does not participate in cytokine secretion (Vemulapalli et al., 2019), but its blockage significantly reduced NO and the pro-inflammatory mediators IL-6 and TNF-α, due to reduced cellular recruitment.

During the silicotic process, injured lung tissue is repopulated with fibroblasts, yielding excessive extracellular matrix deposition and fibrosis, followed by impairment of lung function (Willis and Borok, 2007). In murine models of lung fibrosis, IL-1β has been associated with collagen deposition, while IL-1β receptor blockage reduces pulmonary fibrosis caused by silica or bleomycin (Rimal et al., 2005). IL-6 also promotes pulmonary fibrosis after silica exposure, with excessive extracellular matrix proliferation (Le et al., 2014; Tripathi et al., 2010). In addition, TGF-β is a main pro-fibrotic mediator in remodeling after tissue injury (Fernandez and Eickelberg, 2012), as well as in the fibrotic process trigged by silica exposure (Jagirdar et al., 1996; Moncao-Ribeiro et al., 2014). TGF-β induces extracellular matrix remodeling, collagen production and fibroblast proliferation in the lung parenchyma. Once secreted, TGF-β has chemotactic and proliferative effects on fibroblasts (Sime and O’Reilly, 2001). It also stimulates the secretion of various proinflammatory and fibrogenic cytokines, including TNF-α, IL-13, and IL-1β, thereby increasing and perpetuating the fibrotic response in lung tissue (Fernandez and Eickelberg, 2012). Our results demonstrate that P2Y_12_ inhibition significantly reduces TGF-β production, thus supporting a role for P2Y_12_ signaling in silica-induced fibrosis through TGF-β modulation. Silica exposure impairs lung function – affecting its elastic, resistive and viscoelastic components. Lung functional changes are secondary to granuloma formation, alveolar collapse, as well as cellular infiltration in the lung parenchyma (Faffe et al., 2001; Moncao-Ribeiro et al., 2014; Cruz et al., 2016). Silica particle inhalation also promotes a fibrogenic response characterized by lung remodeling and replacement of damaged epithelial cells with collagen fiber deposition in the lung parenchyma (Honma et al., 2004; Willis and Borok, 2007). P2Y_12_ receptor blockage reduced inflammation and lung remodeling significantly enough to prevent functional changes. Our data corroborate previous observations and expand the understanding of purinergic signaling in silica-induced lung changes. Finally, it is worth note that the animal model of silica-exposure used in the present study does not reproduce chronic silicosis. It does present, however, well-established functional and histological pulmonary changes 14 days after silica administration (Faffe et al., 2001; Borges et al., 2002). Silica-induced chronic lung fibrosis usually results from long-lasting inflammation. Therefore, a better understanding of P2Y_12_ receptor role in acute inflammation would improve our knowledge about purinergic signaling in silicosis, opening new avenues to modify disease progression.

## Conclusion

In conclusion, our results demonstrate that P2Y_12_ receptor is involved in silicosis, probably via its immunomodulatory effects. These findings corroborate and expand previous observations of purinergic signaling participation in silica-induced lung changes. Identification of novel mechanisms involved in disease progression may help in the development of efficient therapies.

## Data Availability Statement

The datasets generated for this study are available on request to the corresponding author.

## Ethics Statement

The animal study was reviewed and approved by the Ethics Committee of the Health Sciences Center, Federal University of Rio de Janeiro (IBCCF164).

## Author Contributions

PS, TL-G, and RC-S drafted the manuscript. MR-F, TL-G, and PS conducted and analyzed the data from all experiments. AT and CD conducted the cytokine measurement experiments. DF, WZ, CT, and RC-S contributed to the conception and design of the study, and revised the draft. All authors contributed to the manuscript revision, and approved the submitted version.

## Conflict of Interest

The authors declare that the research was conducted in the absence of any commercial or financial relationships that could be construed as a potential conflict of interest.
